# Supplementary material for: TRAF6 directs FOXP3 localization and facilitates regulatory T‐cell function through K63‐linked ubiquitination
Source: EMBO J. 2019 Mar 18;38(9):e99766. doi: 10.15252/embj.201899766 (PMC6484404; doi:10.15252/embj.201899766)
Supplement: Supplementary file 1 — Appendix [file EMBJ-38-e99766-s001.pdf]

TRAF6 directs Foxp3 localization and facilitates Treg function through K63-type ubiquitination
APPENDIX

Xuhao Ni[1,3†], Wei Kou[2†], Jian Gu[1†], Ping Wei[3], Xiao Wu[1], Hao Peng[1], Jinhui Tao[3], Wei Yan[1], Xiaoping Yang[3], Andriana Lebid[3], Benjamin V. Park[3], Zuojia Chen[4], Yizhu Tian[1], Juan Fu[3], Stephanie Newman[5], Xiaoming Wang[9], Hongbin Shen[6], Bin Li[4], Bruce R. Blazar[8], Xuehao Wang[1,7], Joseph Barbi[5*], Fan Pan[3*], Ling Lu[1,7*]

[1]Hepatobiliary Center, First Affiliated Hospital, Jiangsu Key Laboratory of Xenotransplantation, Collaborative Innovation Center for Cancer Medicine, Nanjing Medical University, Nanjing, Jiangsu, China
[2]Department of Otolaryngology, The Children's Hospital of Chongqing Medical University, 136[#] Zhongshanger Road, Chongqing, China
[3]Immunology and Hematopoiesis Division, Department of Oncology, Sidney Kimmel Comprehensive Cancer Center, Johns Hopkins University School of Medicine, Baltimore, Maryland, USA
[4]Shanghai Institute of Immunology, Shanghai JiaoTong University School of Medicine, Shanghai, China;
[5]Department of Immunology, Roswell Park Cancer Institute, Buffalo, New York, USA
[6]Department of Epidemiology and Biostatistics, School of Public Health, Collaborative Innovation Center for Cancer Medicine, Nanjing Medical University, Nanjing, Jiangsu, China
[7]State Key Laboratory of Reproductive Medicine, Nanjing Medical University, Nanjing, Jiangsu, China;
[8]Department of Pediatrics, Division of Blood and Marrow Transplantation, University of Minnesota, Minneapolis, MN, USA
[9]Department of Immunology, State Key Laboratory of Reproductive Medicine, Nanjing Medical University, Nanjing, China

† These authors contributed equally to this work.
[*]To whom correspondence may be addressed. Email Address correspondence to: lvling@njmu.edu.cn, fpan1@jhmi.edu or joseph.barbi@roswellpark.org.

Running title: TRAF6 controls Foxp3 by K63 modification

TABLE OF CONTENTS:

Appendix Figure S1
Appendix Figure S1 Legends
Appendix Figure S2
Appendix Figure S2 Legends
Appendix Figure S3
Appendix Figure S3 Legends
Appendix Figure S4
Appendix Figure S4 Legends
Appendix Table S1
Appendix Table S1 Legends

Appendix Figure S1

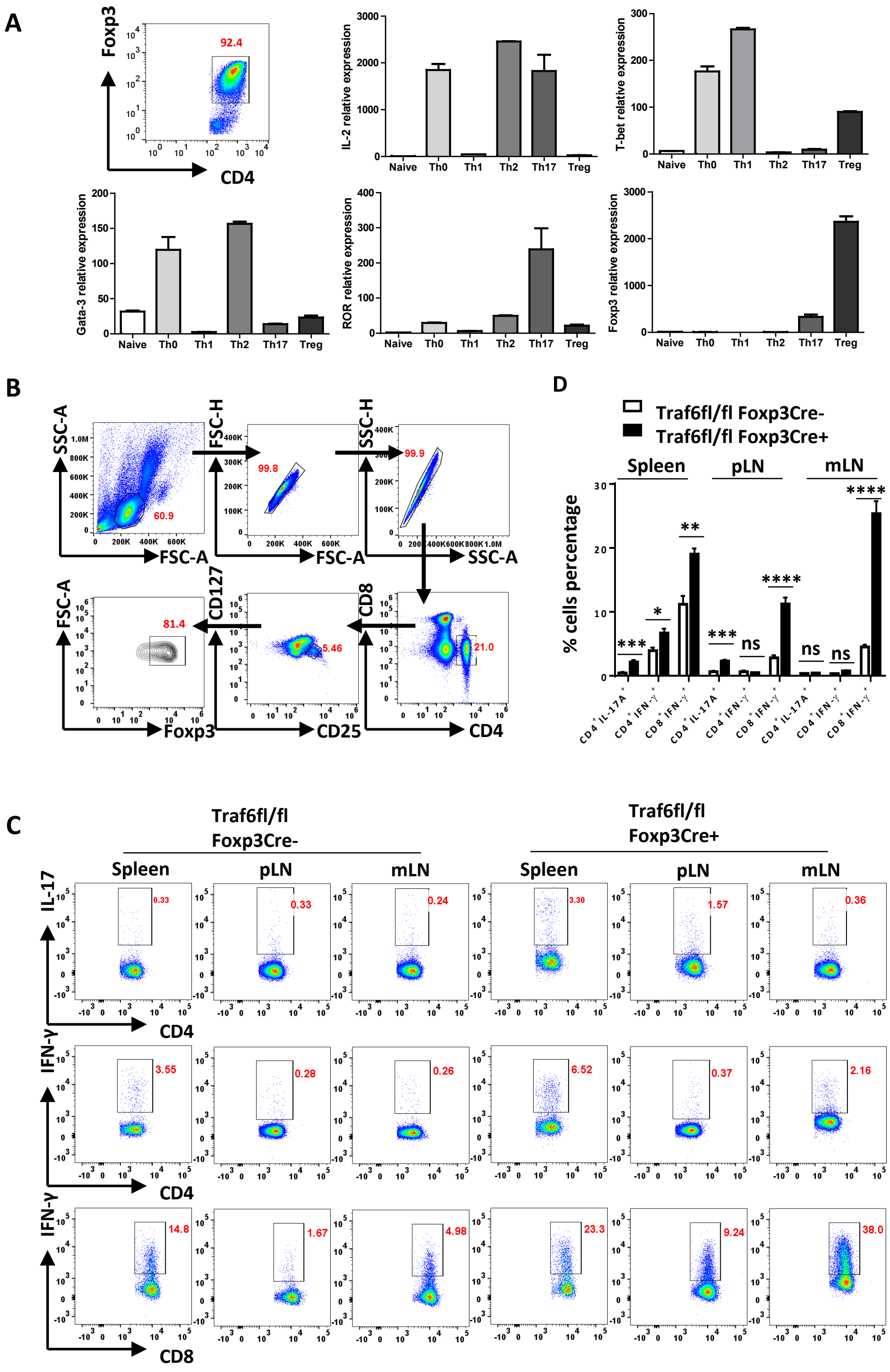

Appendix Figure S1. In vitro T cell differentiation, and representative FOXP3 staining, and proinflammatory cytokine production by the T cells of mice with TRAF6-deficient Tregs.

(A, upper left) Representative results of in vitro iTreg generation. Naïve CD4+ T cells were obtained from wild type C57BL/6 mice by FACS and activated with anti-CD3/CD28 (1μg, and 2μg/ml) in the presence of iTregs inducing cytokines (100U/ml IL-2, 5ng/ml TGFβ). After 72 hours, cells were harvested and intracellular FOXP3 was detected by immunostaining and flow cytometry analysis. (A upper center, lower panels) the levels of Il2, Tbx21 (Tbet), Gata3, RORc (RORγt) and Foxp3 transcript in different T helper cell subsets. CD4+ Naïve T cells isolated from wild type C57BL/6 mice were induced to different T helper cell subsets in vitro. (B) Representative flow cytometry gating strategy and purity of isolated peripheral blood Tregs from human blood donors. (C, D) Frequencies of proinflammatory cytokine-producing T cells in mice with Treg-specific TRAF6 deficiency and wild type controls. Suspensions of leukocytes from the spleens, peripheral lymph nodes (pLN) and mesenteric lymph nodes (mLN) were obtained from the tissues of Traf6fl/flFoxp3Cre+ and wild type (Traf6fl/flFoxp3Cre-) mice. Cells were stimulated ex vivo with PMA and ionomycin in the presence of Golgistop for 5 hours. Surface markers CD4 and CD8 were stained prior to fixation, permeabilization (BD), and intracellular cytokine staining. Frequencies of the producers of the indicated cytokines within CD4+ and CD8+ T cells were found by flow cytometry (5 mice/group). *P<0.05; **P<0.01; ***P<0.001; ****P<0.0001; ns, no significance (unpaired t test). Data represent the mean findings of at least 3 independent experiments +/-SEM, except panels A (upper left), B, and C, which depict representative findings.

Appendix Figure S2

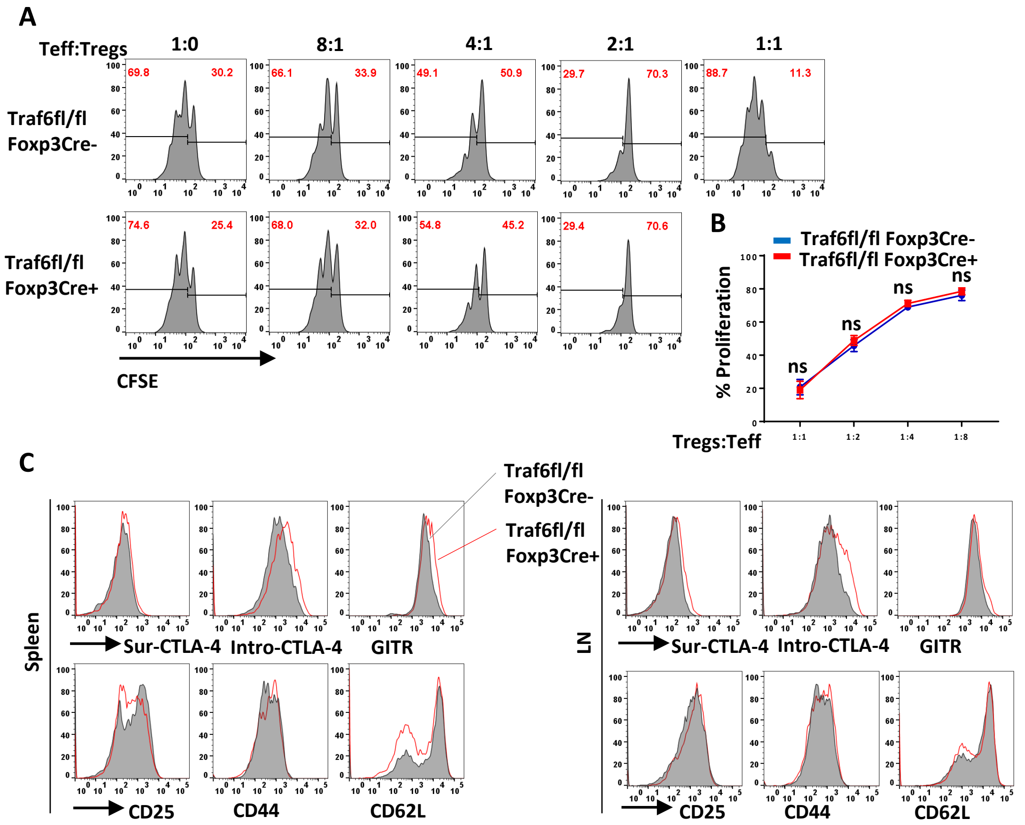

Appendix Figure S2. Treg-specific TRAF6 deficiency does not markedly alter in vitro Treg function but alters expression of some Treg-associated factors expression and reduces K63 ubiquitination of FOXP3.

(A, B) In vitro suppressive potency of Tregs isolated from wild type (Traf6fl/flFoxp3Cre-) and Traf6fl/flFoxp3Cre+ mice. Tregs from the indicated mice were isolated by FACS, as were naïve CD4+ (CD62Lhigh/CD25-) responder T cells. Responder cells were stained with CFSE and co-cultured with Tregs at varying ratios in the presence of anti-CD3/CD28 antibodies. The extent of responder cell proliferation (dilution of CFSE signal) was assessed by flow cytometry. (C) Expression of Treg-associated factors including GITR, CD25, as well as surface ("sur") and intracellular ("intro") CTLA-4 by FOXP3+/CD4+ cells of Traf6fl/flFoxp3Cre+ mice Tregs and their wild type (Traf6fl/flFoxp3Cre-) littermates was measured by flow cytometry. CD44 and CD62L, makers of activation and resting states, respectively, were also assessed. Shown are representative findings from at least three experiments. ns, no significance (unpaired t test). Panel B depicts mean +/-SEM.

Appendix Figure S3

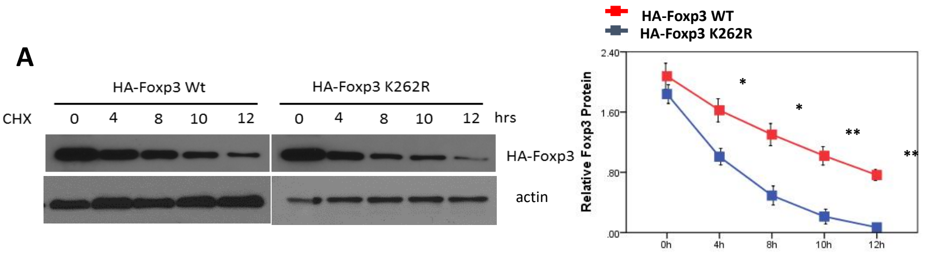

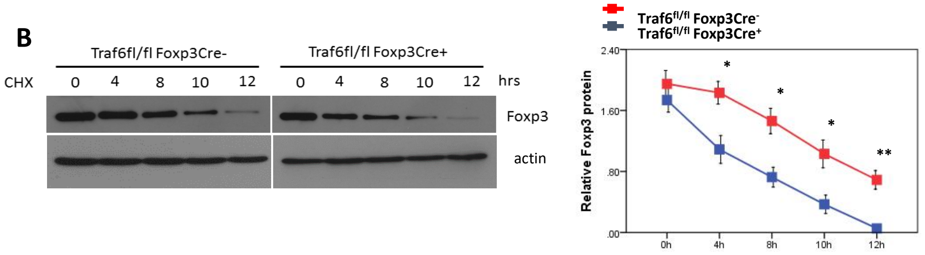

Appendix Figure S3. Down-modulating TRAF6 activity and expression adversely affects FOXP3 protein stability.

(A) Stability of the FOXP3 protein pool in the absence of K262 ubiquitination. 293T cells carrying an expression vector encoding either wild type FOXP3 or the K262R lysine mutant were treated with 5µg/ml cyclohexamide (CHX) for the indicated periods. The relative turnover of the FOXP3 protein pool in each group was observed by lysing the cells for immunoblot analysis. (B) FOXP3 half-life in murine Tregs with or without TRAF6 expression. Tregs were isolated from Traf6fl/flFoxp3Cre+ and Traf6fl/flFoxp3Cre- (wild type) mice and treated with CHX for the indicated time. FOXP3 levels were measured by Immunoblot analysis. Immunoblots depicted in panel A and B are representative findings from at least three independent experiments and the accompanying quantitation shows the mean FOXP3 band densities across these experiments +/-SEM.

Appendix Figure S4

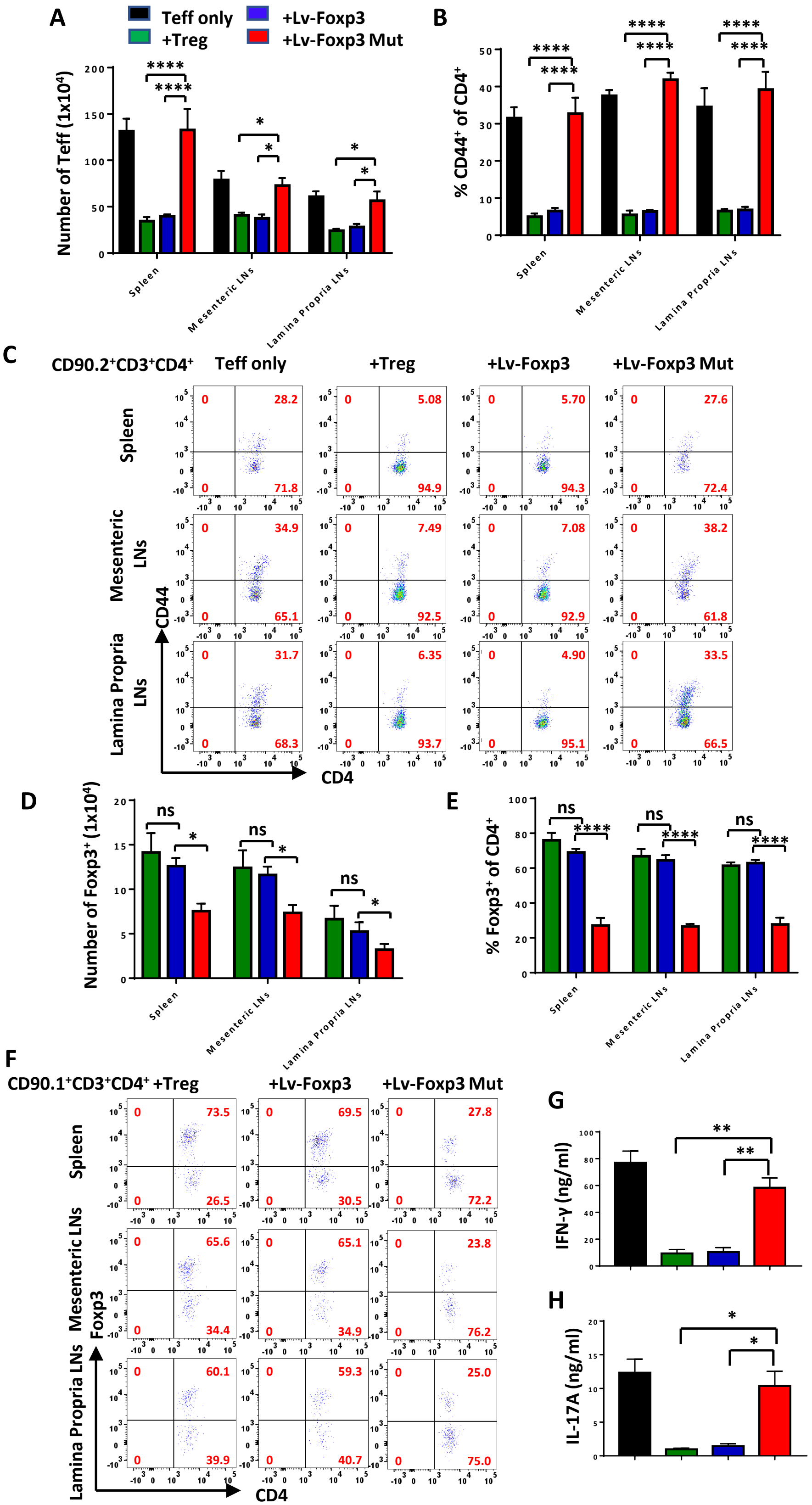

Appendix Figure S4. Absence of K262 ubiquitination impairs FOXP3 expression, and suppressive function, but not cellular fitness in vivo.

Characterizing effector and regulatory T cell populations in the presence or absence of Tregs capable of K262 FOXP3 ubiquitination. Naïve CD4+ T cells were purified from Thy1.1/CD90.1+ BALB/c mice and subjected to retroviral transduction to express either wild type Foxp3 or a K262R mutant resistant to ubiquitination that lysine residue 226. These cells were co-injected into Rag2- /- mice along with Thy1.2/CD90.2+ naïve CD4+ T cells as in Fig. 6. 8 weeks later, the spleen-, mesenteric lymph node- and lamina propria-infiltrating leukocytes were recovered and characterized by flow cytometry. (A) The numbers of Thy1.2/CD90.2+ T effectors (Teff) recovered from the indicated tissues of recipient mice were found. (B and C) The frequencies of Thy1.2/CD90.2+CD3+CD4+CD44+ Teff were assessed. (D, E and F) Similarly, the number and frequencies of FOXP3-expressing cells within the transferred “Treg” population (Thy1.1/CD90.1+) were assessed. (G and H) Proinflammatory cytokines IFNγ and IL-17 production by CD4+ lamina propria lymphocytes was analysed by ELISA. For A, B, D, E, G, and H, shown are the mean numbers of cells (+/-SEM) recovered from 6 mice per group over three independent experiments. *$P<0.05$; **$P<0.01$; ****$P<0.0001$; ns, no significance, two-way ANOVA.

Appendix Table S1

Antibody name	Clone	Cat#	Company
Anti-mouse Alexa Fluor 488 -Foxp3	MF-14	126406	Biolegend
Anti-mouse/Rat PE-Foxp3	FJK-16s	72577540	eBioscience
Anti-mouse APC-IFN-γ	XMG1.2	505810	Biolegend
Anti-mouse FITC-IL-17A	eBio17B7	11717781	eBioscience
Anti-mouse BV-BV750-TNF-α	MAb11	566359	BD Biosciences
Anti-mouse Pacific Blue-CD4	RM4-5	558107	BD Biosciences
Anti-mouse BV510-CD8	53-6.7	563068	BD Biosciences
Anti-mouse AF700-CD3	17A2	100216	Biolegend
Anti-mouse PerCP-Cy5.5-CD45.1	A20	560580	BD Biosciences
Anti-mouse APC-CD45.2	104	109814	Biolegend
Anti-mouse FITC-CD44	IM7	11044181	eBioscience
Anti-mouse PE-Cy7-CD62L	MEL-14	560516	BD Biosciences
Anti-mouse PE-CTLA-4	UC104B9	12152282	eBioscience
Anti-mouse APC-GITR	DTA-1	126312	Biolegend
Anti-mouse BV605-CD25	PC61	563061	BD Biosciences
Anti-human FITC-CD127	HIL-7R-M21	560549	BD Biosciences
Anti-human PE-CD25	BC96	302606	Biolegend
Anti-human PerCP-Cy5.5-CD8	SK1	565310	BD Biosciences
Anti-human APC-CD4	RPA-T4	300537	Biolegend
Anti-HA	C29F4	3724S	Cell Signaling Technology
Anti-FLAG	D6W5B	14793S	Cell Signaling Technology
Anti-Myc	9B11	2276S	Cell Signaling Technology
Anti-Foxp3		ab75763	Abcam
Anti-Traf6	D21G3	8028S	Cell Signaling Technology
Anti-actin	8H10D10	3700S	Cell Signaling Technology

Appendix Table S1. Summary of the clones and vendors for the antibodies used in this study.
