# Supplementary material for: TRAF6 directs FOXP3 localization and facilitates regulatory T‐cell function through K63‐linked ubiquitination
Source: EMBO J. 2019 Mar 18;38(9):e99766. doi: 10.15252/embj.201899766 (PMC6484404; doi:10.15252/embj.201899766)
Supplement: Supplementary file 2 — Expanded View Figures PDF [file EMBJ-38-e99766-s002.pdf]

Expanded View Figures

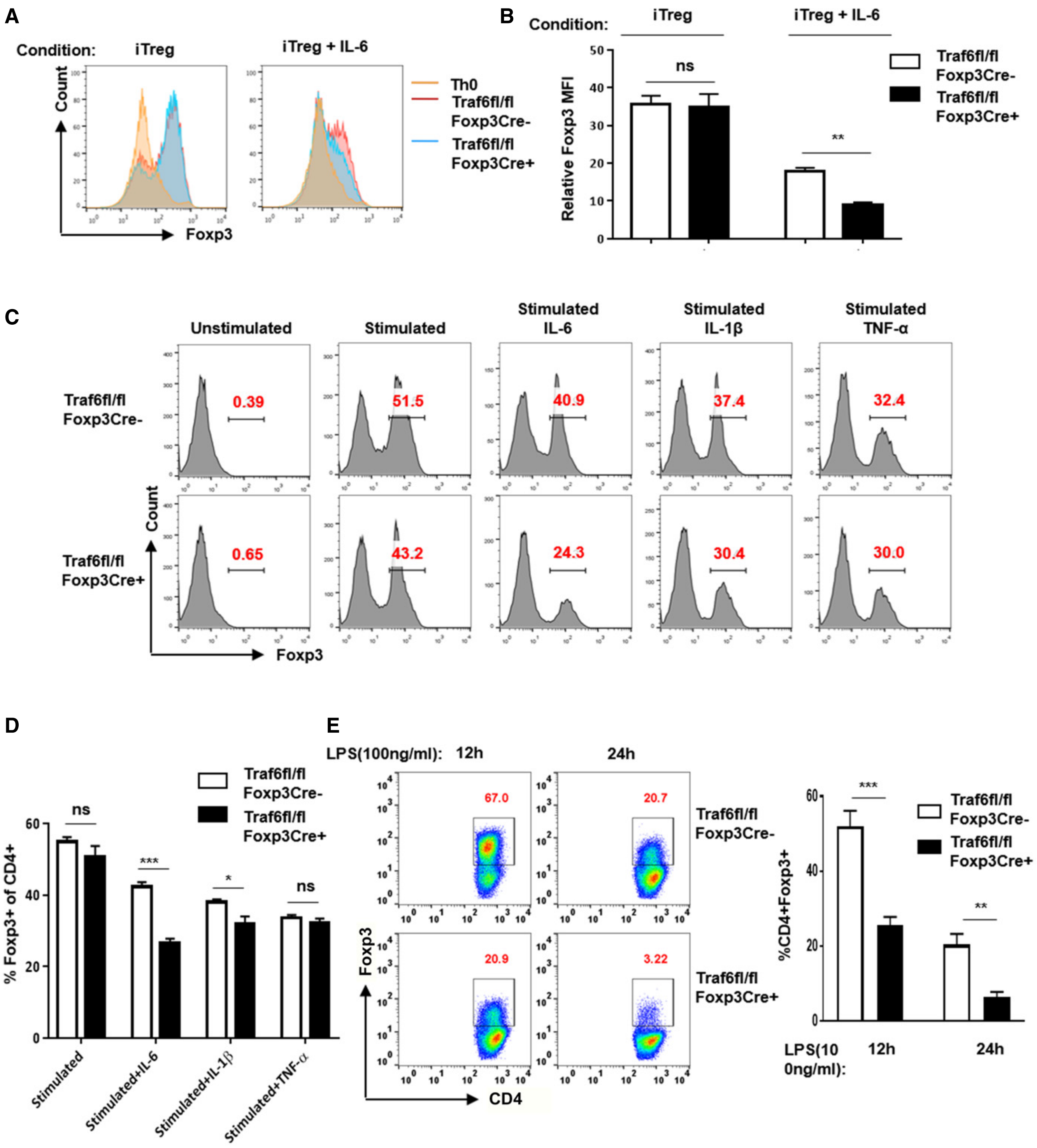

Figure EV1.

◀ **Figure EV1. Treg-specific TRAF6 deficiency exacerbates the disruption of FOXP3 expression by iTreg exposed to inflammatory cues.**

A, B Impact of TRAF6 deficiency on iTreg destabilization. Naïve $CD4^+$ T cells were FACS purified from the lymph nodes and spleens of $Traf6^{fl/fl}Foxp3Cre^+$ mice and wild-type ($Traf6^{fl/fl}Foxp3Cre^-$) mice and activated *in vitro* in the presence of FOXP3-inducing cytokines (TGFβ and IL-2) for 72 h. Expression of FOXP3 under these iTreg-skewing conditions and upon addition of IL-6 (20 ng/ml) to the media was determined by intracellular staining followed by flow cytometry.

C, D FOXP3 expression in the presence or absence of TRAF6 and proinflammatory cytokines. Expression of FOXP3 by naïve $CD4^+$ T cells at baseline, under iTreg-skewing conditions and upon addition of IL-6 (20 ng/ml), IL-1β (20 ng/ml) and TNF-α (100 ng/ml), to the media was determined by intracellular staining followed by flow cytometry.

E After deriving iTregs *in vitro* from naïve precursors as described above, iTregs were incubated with LPS (100 ng/ml) for 12 or 24 h. Expression of FOXP3 was measured by intracellular staining followed by flow cytometry.

Data information: Panel (B, D and E right) show mean ± SEM (3–5 replicates each). (A, C and E left) depict representative results from three experiments. *$P < 0.05$; **$P < 0.01$; ***$P < 0.001$; ns, no significance (unpaired *t*-test).

© 2019 The Authors

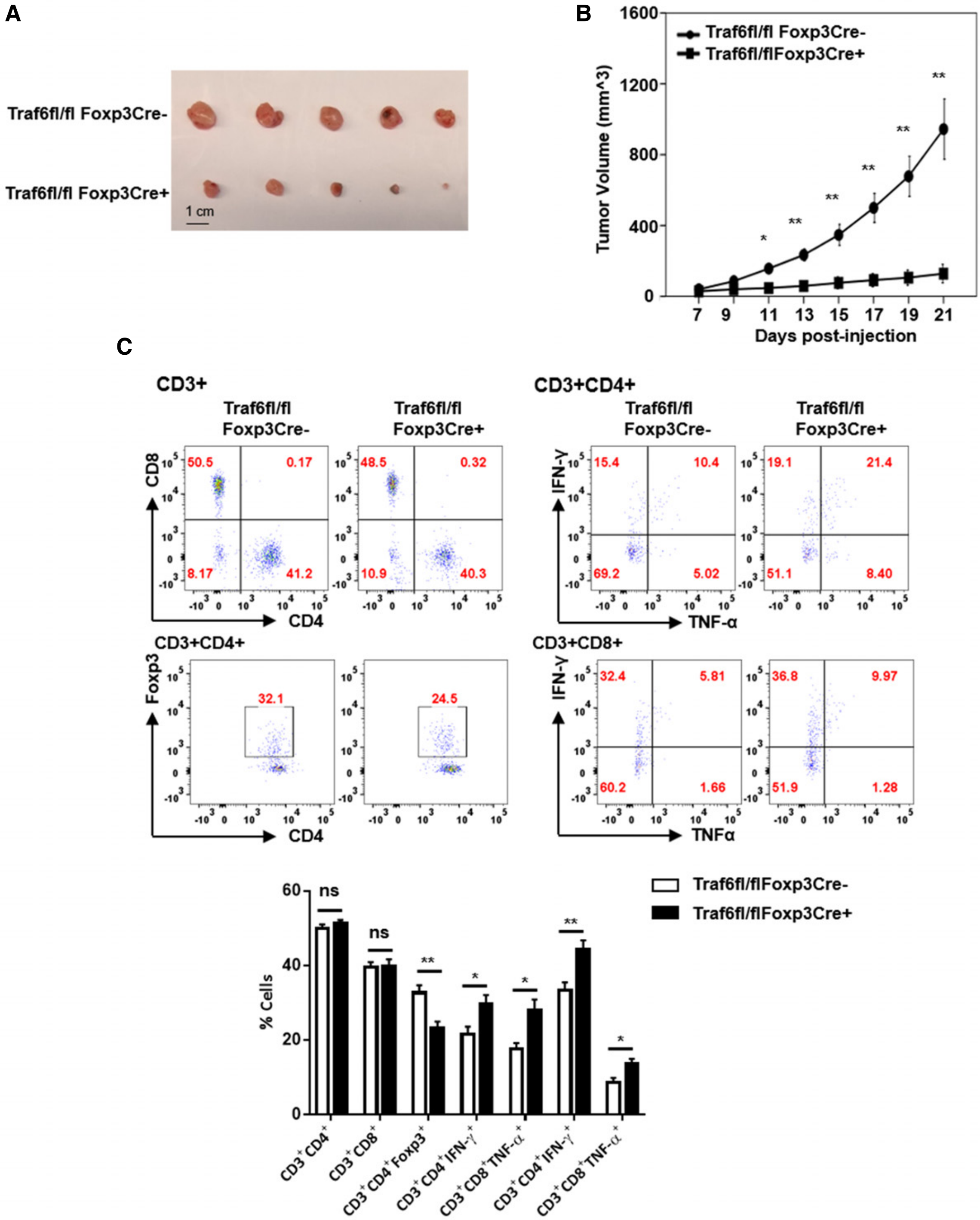

Figure EV2. Treg-specific TRAF6 deficiency delays the growth of MC38 colon carcinoma cells while enhancing indicator anti-tumor immunity.

A, B The growth of implanted MC38 tumors in Traf6$^{fl/fl}$Foxp3Cre^{+} mice and Traf6$^{fl/fl}$Foxp3Cre^{-} (wild type) littermates. 1×10^5 MC38 cells passaged *in vitro* were injected s.c. into the shaved flanks of the indicated mice (n = 5/group). Tumor volumes were monitored every 2–3 days (Scale bars: 1 cm).

C Flow cytometric characterization of tumor-infiltrating leukocytes from mice in (A and B). Suspensions of leukocytes recovered from the indicated tumor-bearing mice were stained for surface markers (CD3, CD4, CD8) and intracellular FOXP3 or proinflammatory cytokines (following *ex vivo* reactivation in the presence of PMA/ionomycin and Golgi Stop, fixation, and permeabilization). Cell frequencies within the indicated populations are shown (five replicates).

Data information: (A and C left) depict representative findings from two independent experiments, while (B and C right) show mean tumor volumes over time and cell frequencies ± SEM from a representative experiment. *P < 0.05; **P < 0.01 (unpaired Student's t-test).

© 2019 The Authors

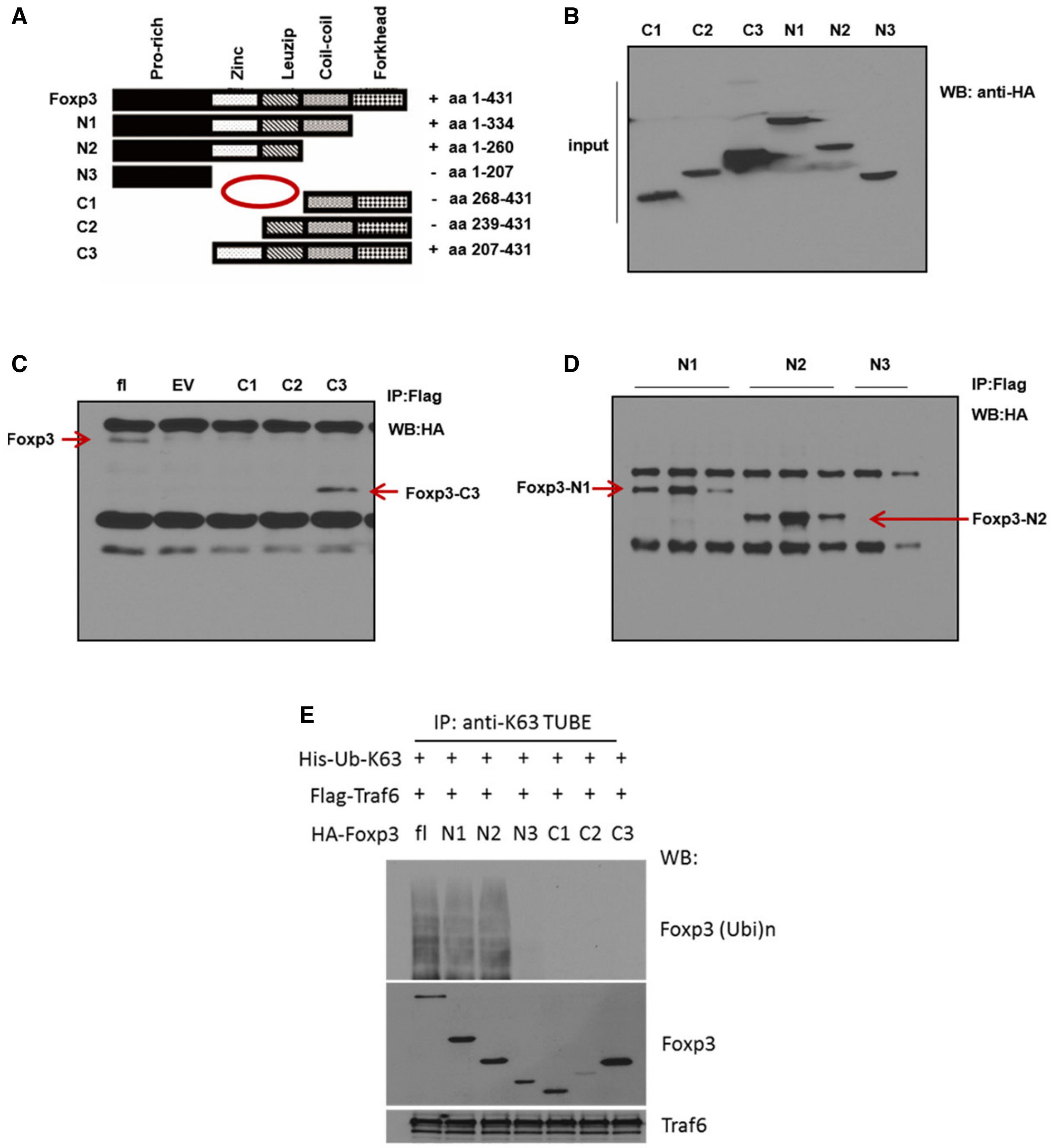

Figure EV3. The zinc finger and leucine zipper domains of FOXP3 are necessary for interaction with TRAF6 and K63 ubiquitination.

A Schema for generating deletion mutant constructs encoding HA-labeled FOXP3 species lacking specific FOXP3 regions.

B 293T cells were transfected with the individual deletion mutant constructs, and immunoblotting of cell lysates for HA confirmed the predicted sizes of FOXP3 proteins lacking specified domains.

C, D Each deletion mutant as well as a full-length FOXP3 encoding construct and an empty vector control was then co-expressed with FLAG-tagged TRAF6 molecules in 293T cells. Pull-down with anti-FLAG beads and subsequent immunoblotting for HA revealed which FOXP3 variants could interact with TRAF6.

E Assessment of K63 ubiquitination among *Foxp3* deletion mutant gene products. Following lysis of cells carrying the indicated combinations of TRAF6-FLAG and HA-FOXP3 deletion mutant encoding constructs, levels of K63-ubiquitinated FOXP3 species in each were determined by pulling down K63-ubiquitinated proteins using bead-immobilized K63 TUBE reagent and immunoblot probing for FOXP3 among the immunoprecipitated proteins. The expression of FOXP3 and TRAF6 by transfectants (input) in these studies was also confirmed by immunoblot analysis.

Data information: Shown are representative blots from three experiments.

 © 2019 The Authors

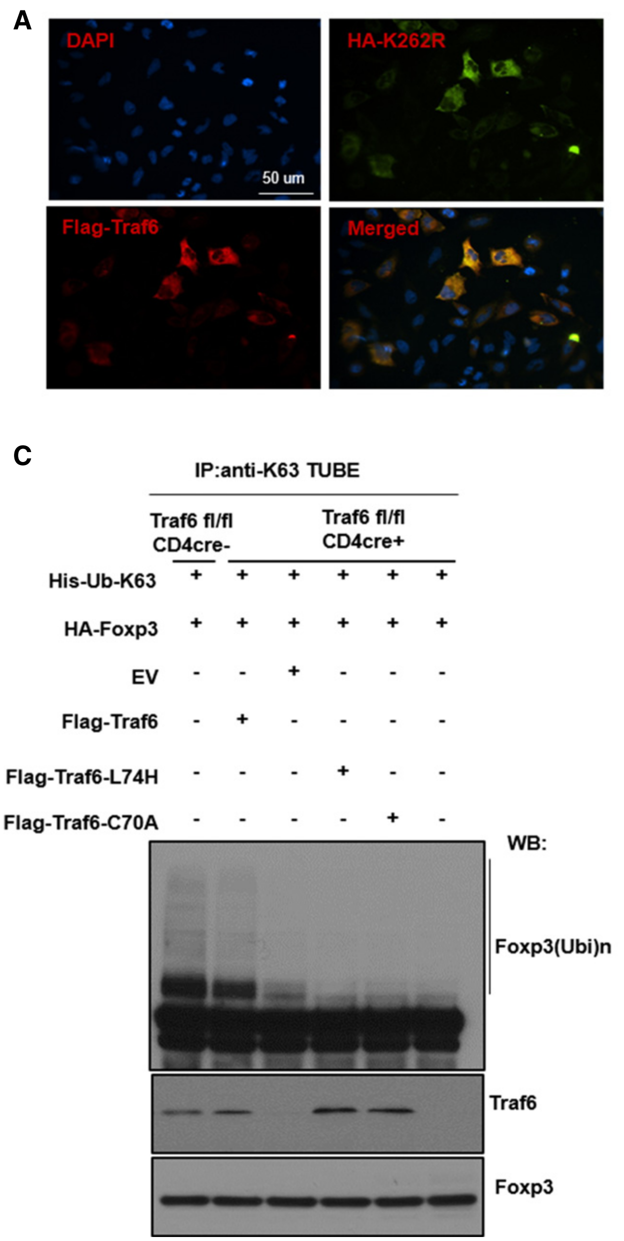

Figure EV4. Mutation at FOXP3 residue K262 does not prevent association with TRAF6, and enzymatically defective TRAF6 mutants recapitulate the K262 phenotype.

A HeLa cells were co-transfected with expression constructs encoding FLAG-TRAF6 and HA-K262R FOXP3. Cells were processed as described in Materials and Methods and stained with Alexa Fluor 488-labeled anti-HA to visualize K262R FOXP3 and Cy5-labeled anti-FLAG to detect TRAF6. The degree of overlap in the fluorescent signals for these proteins was visualized by fluorescence microscopy, and DAPI was used to mark nuclei. Representative (40×) micrographs for each group are shown.

B Interaction of TRAF6 with wild-type FOXP3 and the K262R mutant. 293T cells were transduced with expression vectors encoding the indicated factors before lysis and reciprocal co-IP. TRAF6 and FOXP3 (wild type and K262R) levels in the precipitated proteins were determined by immunoblot analysis after SDS–PAGE resolution.

C K63 ubiquitination of FOXP3 in TRAF6-deficient CD4$^+$ T cells ectopically expressing wild-type and enzymatically defective TRAF6 mutants. Murine CD4$^+$ T cells lacking TRAF6 (FACS purified from Traf6$^{fl/fl}$CD4Cre$^+$ mice) were reconstituted with retroviral expression vectors encoding wild-type FOXP3 and either the L74H-TRAF6 and C70A-TRAF6 mutant or wild-type TRAF6. The levels of K63-specific FOXP3 ubiquitination in these cells were determined as in Fig 3E.

D Suppression of IL-2 production by wild-type and enzymatic TRAF6 mutants. CD4$^+$ T cells from Traf6$^{fl/fl}$CD4Cre$^+$ mice and Traf6$^{fl/fl}$CD4Cre$^-$ mice were reconstituted as described in (C). Cells were activated as in Fig 5B, and levels of IL-2 production were determined by ELISA of supernatant.

Data information: Depicted in panels (A, B and C) are representative findings from three independent experiments. Panel (D) shows the mean results ± SEM from at least two independent experiments. **$P < 0.01$ (unpaired Student's *t*-test).
Source data are available online for this figure.

© 2019 The Authors

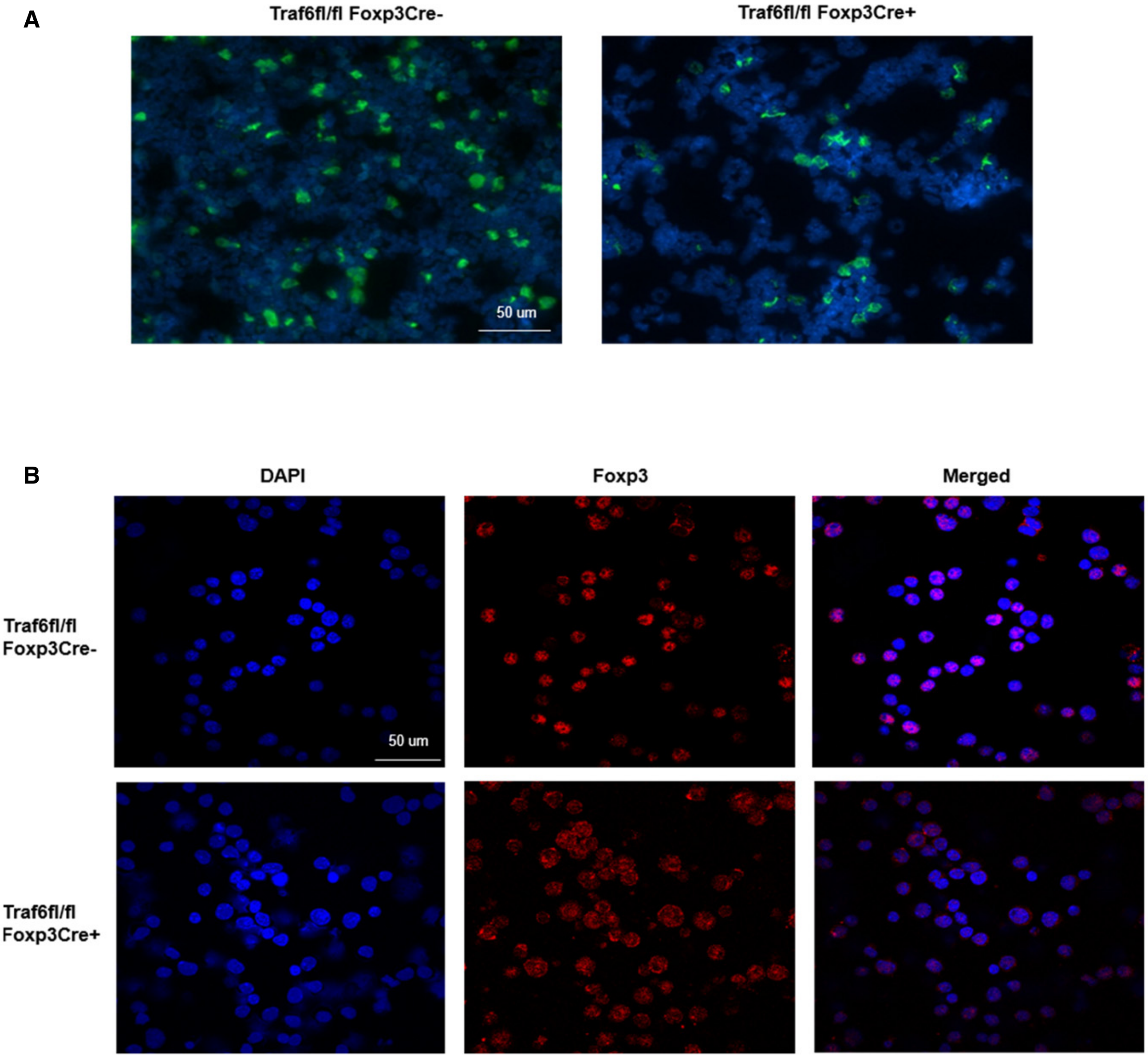

Figure EV5. Perinuclear accumulation of FOXP3 occurs in tumor-bearing mice lacking Treg-specific TRAF6 expression and TRAF6-deficient iTregs.

A Aberrant FOXP3 staining patterns in tumor-bearing mice lacking Treg-specific TRAF6. 1×10^5 B16 melanoma cells were injected s.c. into the shaved flanks of Traf6$^{fl/fl}$Foxp3Cre$^-$ (wild type) and Traf6$^{fl/fl}$Foxp3Cre$^+$ mice. Approximately 21 days later, splenocytes were harvested, FOXP3 protein was detected by immunostaining, and cells were affixed to microscope slides by Cytospin. The distribution of FOXP3 signal relative to nuclei (DAPI$^+$) was observed by immunofluorescence microscopy (blue: DAPI, green: FOXP3, Scale bars: 50 μm).

B Cellular localization of FOXP3 protein in iTregs generated from Traf6$^{fl/fl}$Foxp3Cre$^+$ and Traf6$^{fl/fl}$Foxp3Cre$^-$-derived T cells. Tregs were generated by *in vitro* activation (with anti-CD3/CD28 antibodies; 1 μg and 2 μg/ml, respectively) of FACS purified naïve CD4$^+$ T cells under iTreg-skewing conditions (100 U/ml IL-2 and 5 ng/ml TGFβ) for 4 days. Intracellular FOXP3 was detected by immunostaining as in (A). The degree of colocalization between FOXP3 protein and DAPI$^+$ nuclei was observed by confocal microscopy (blue: DAPI, red: FOXP3, Scale bars: 50 μm).

Data information: Shown are representative blots from three experiments.

 © 2019 The Authors
