# Supplementary figures and images for: TRAF6 directs FOXP3 localization and facilitates regulatory T‐cell function through K63‐linked ubiquitination
Source: EMBO J. 2019 Mar 18;38(9):e99766. doi: 10.15252/embj.201899766 (PMC6484404; doi:10.15252/embj.201899766)

Figure EV4

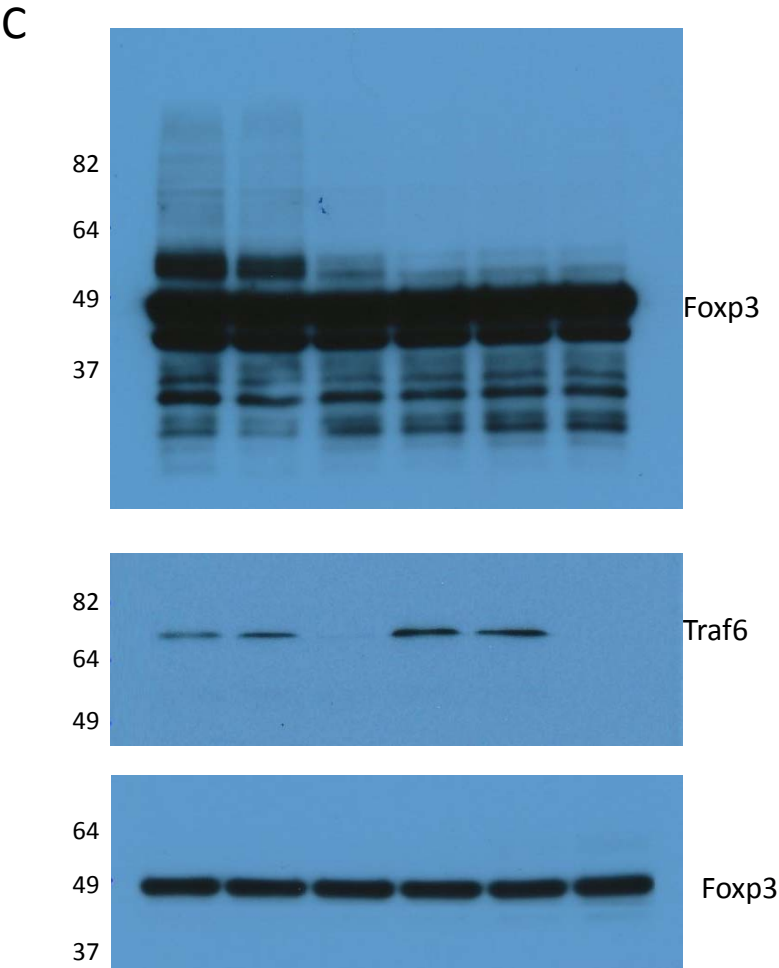

Figure EV4

D

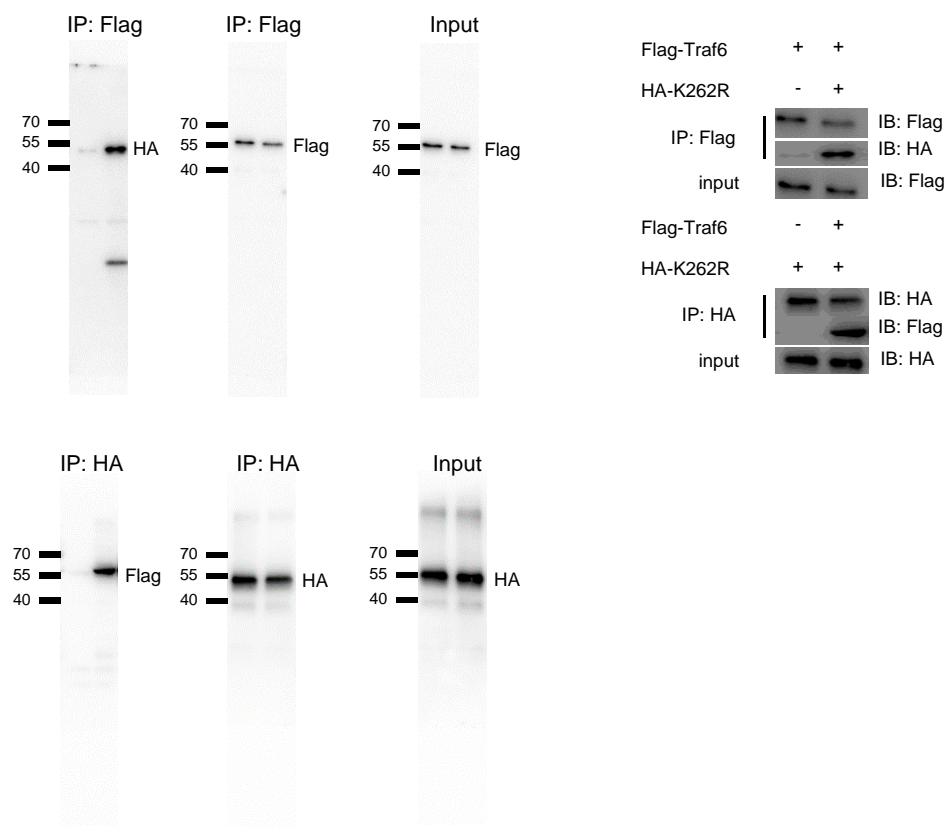

Supplement: Supplementary file 3 — Source Data for Expanded View and Appendix [file EMBJ-38-e99766-s005.zip › Source_Data_for_EV4.pdf]

Figure S3

A

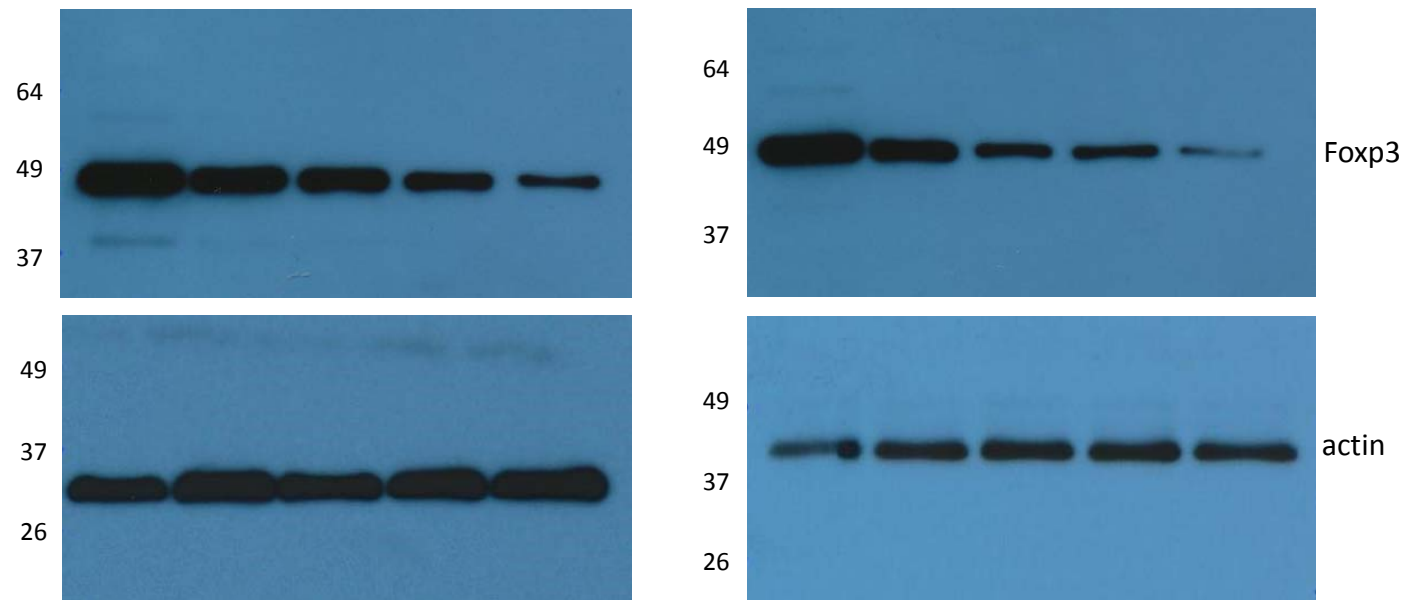

Figure S3

B

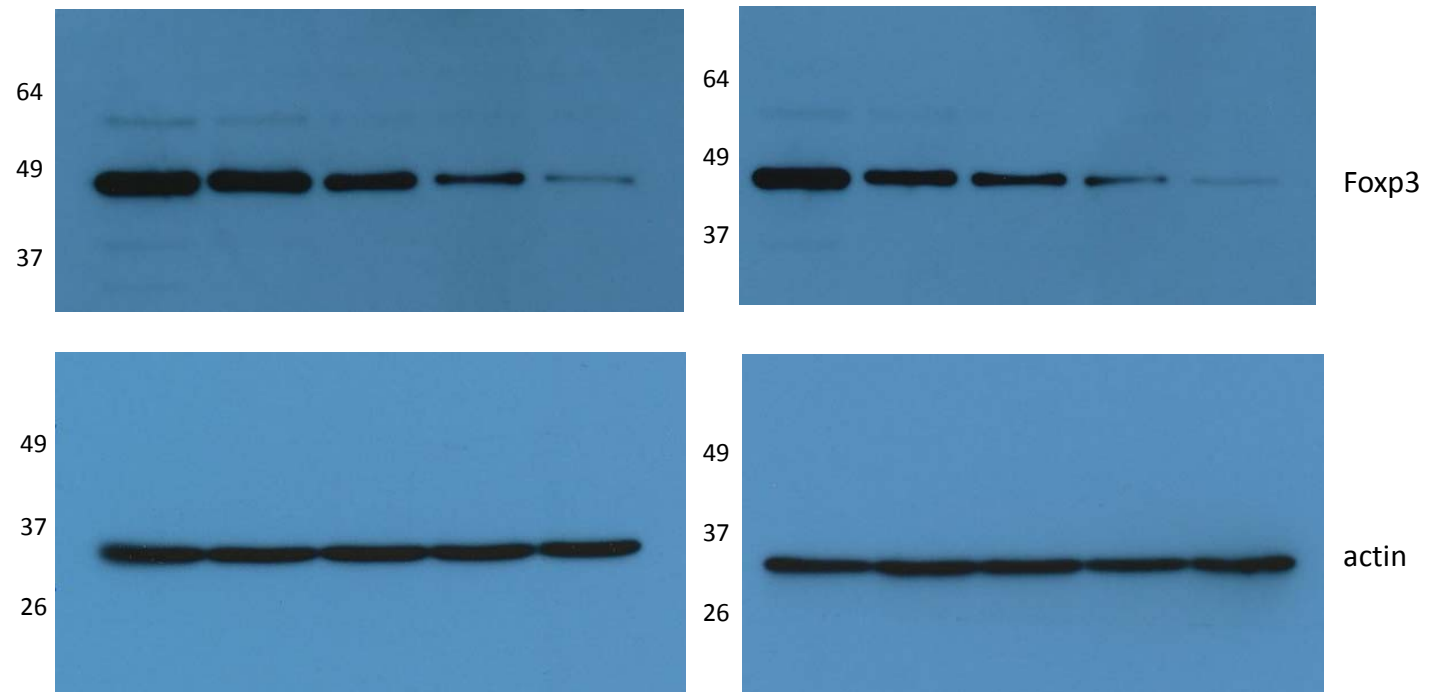

Supplement: Supplementary file 3 — Source Data for Expanded View and Appendix [file EMBJ-38-e99766-s005.zip › SDataAppendixFigs/Source_Data_for_Appendix_S3.pdf]

Figure 3A

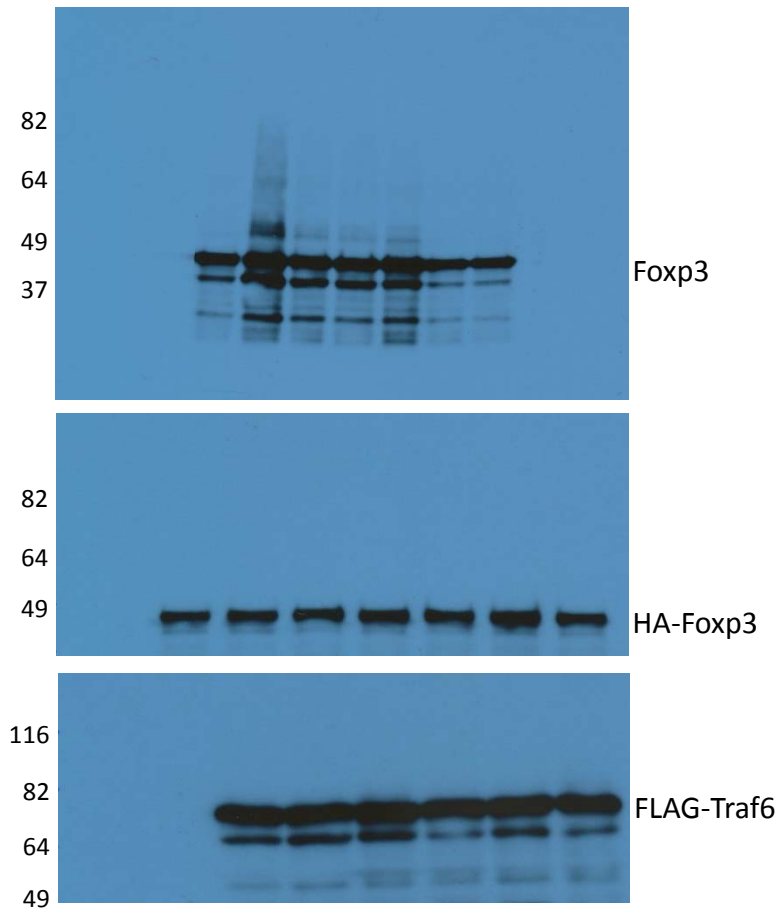

Figure 3C

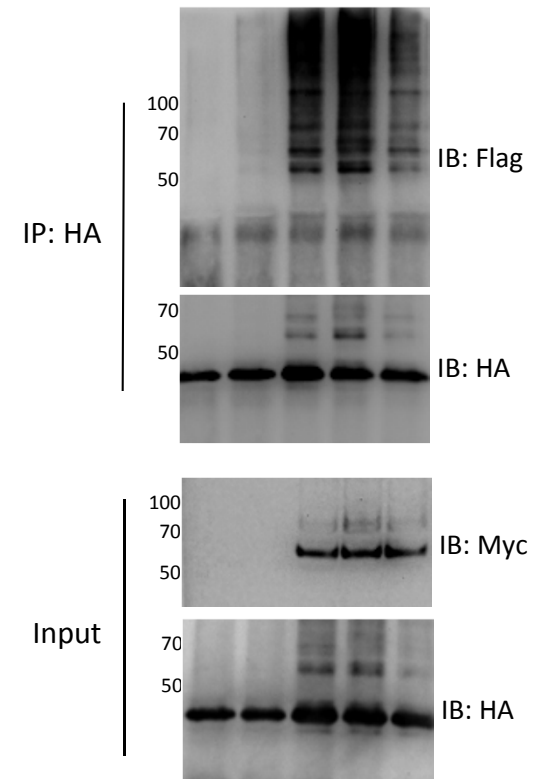

Figure 3D

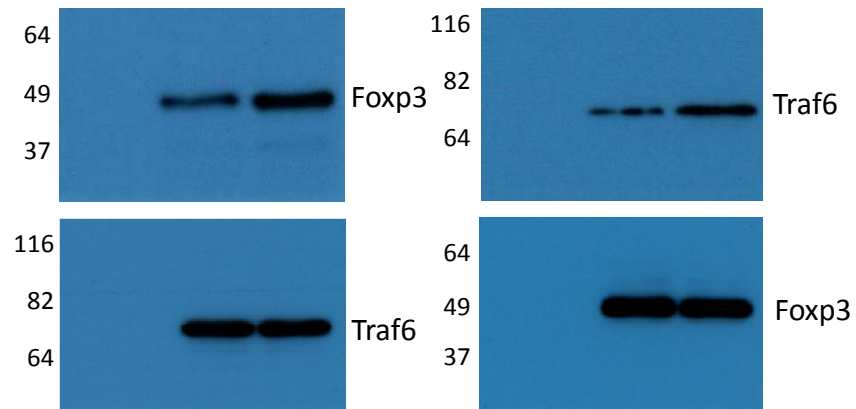

Figure 3E

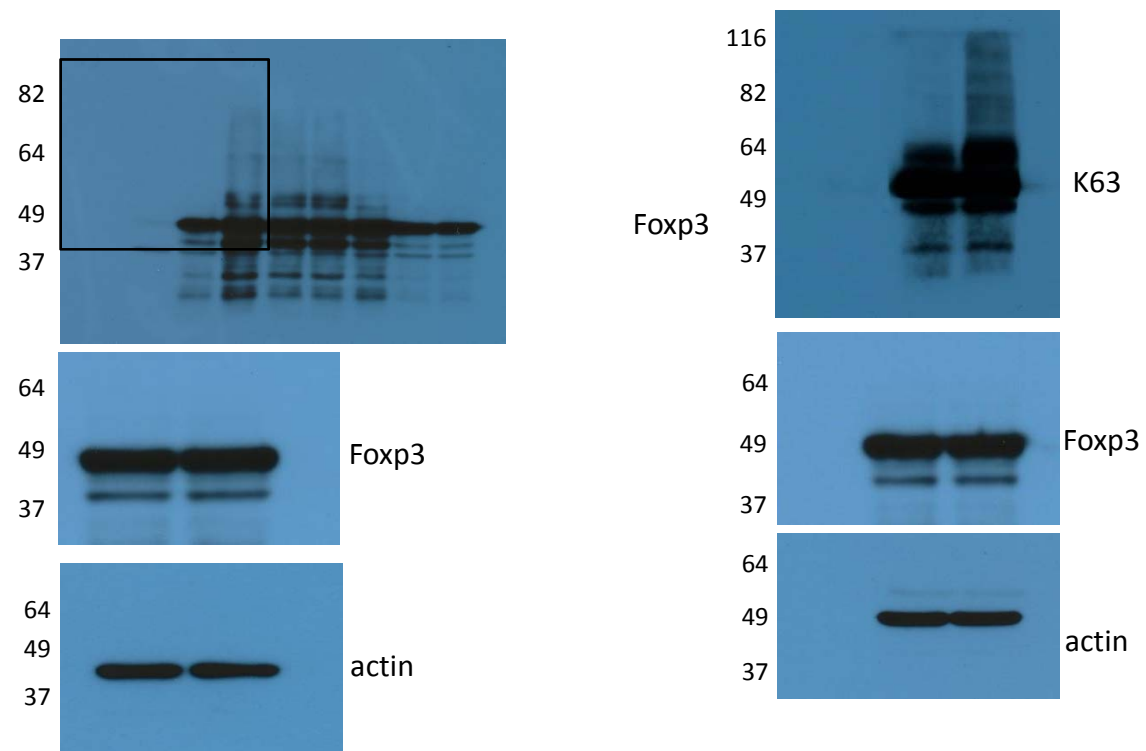

Supplement: Supplementary file 5 — Source Data for Figure 3 [file EMBJ-38-e99766-s003.pdf]

Figure 4A

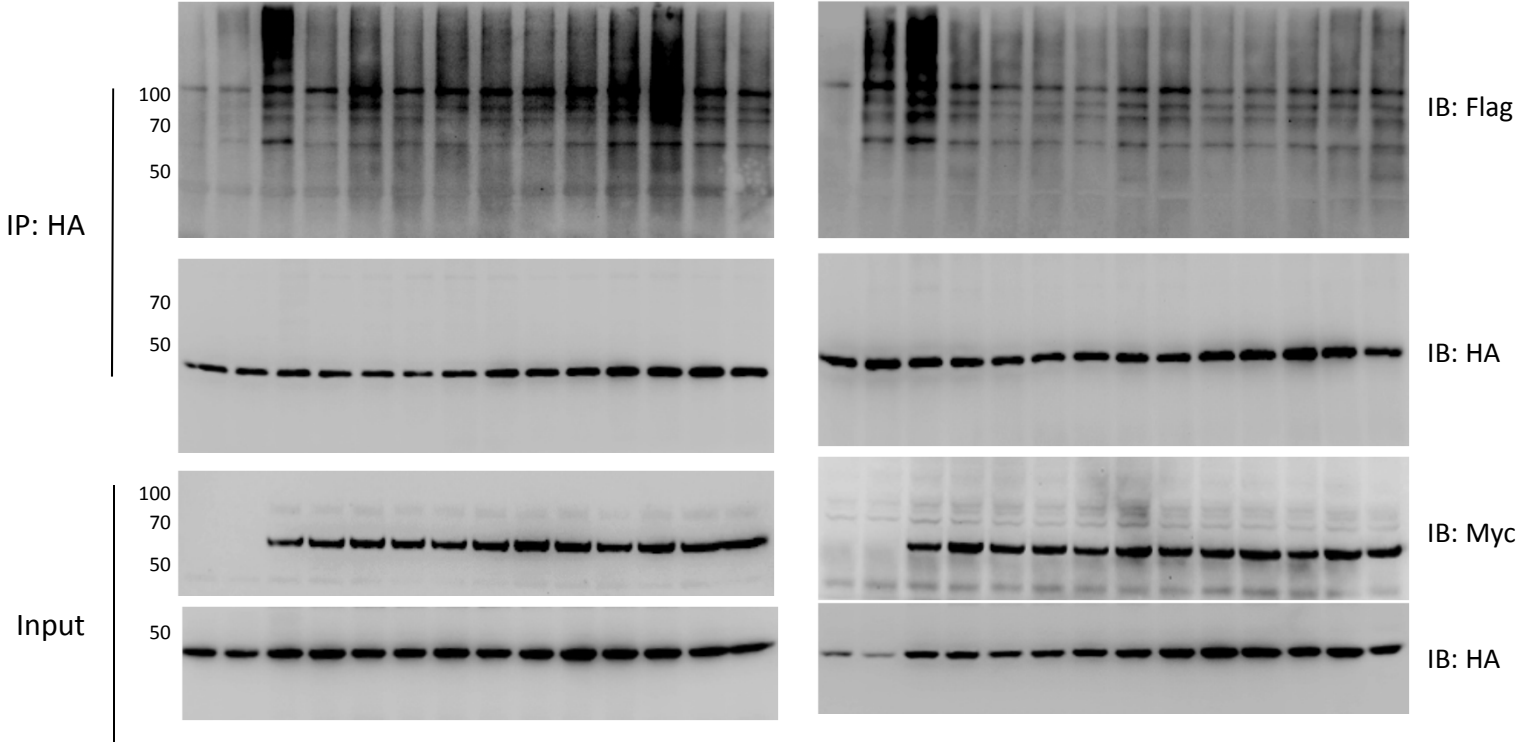

Figure 4B

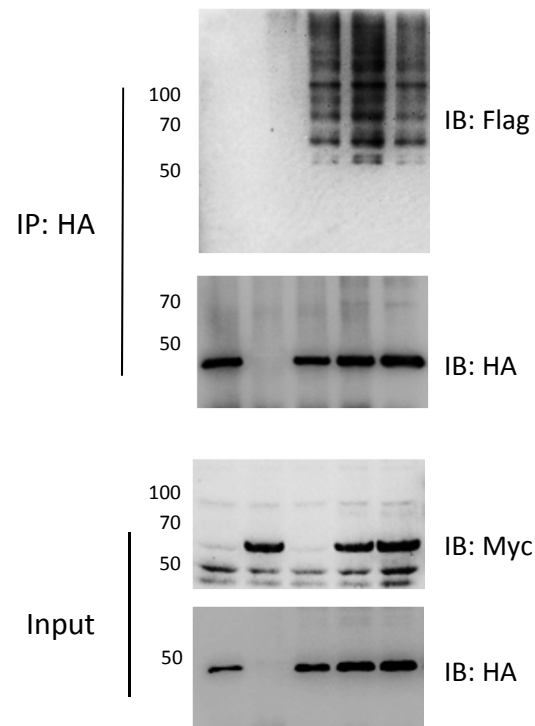

Supplement: Supplementary file 6 — Source Data for Figure 4 [file EMBJ-38-e99766-s004.pdf]
